# Expression of transforming growth factor beta-1 in gastric cancer and in the gastric mucosa of first-degree relatives of patients with gastric cancer

**DOI:** 10.1054/bjoc.1999.1107

**Published:** 2000-06

**Authors:** M P A Ebert, J Yu, S Miehlke, G Fei, U Lendeckel, K Ridwelski, M Stolte, E Bayerdörffer, P Malfertheiner

**Affiliations:** Departments of Gastroenterology, Hepatology and Infectious Diseases, Surgery, Institute of Experimental Internal Medicine, Otto-von-Guericke University Magdeburg, Magdeburg, D-39120, Germany; Medical Department I, Technical University of Dresden, Dresden, D-01307, Germany; Department of Pathology, Klinikum Bayreuth, Bayreuth, D-95445, Germany

**Keywords:** risk, screening, carcinogenesis, stomach, family

## Abstract

Transforming growth factors beta (TGF-β) constitute a family of polypeptide growth factors that control cell growth, cell differentiation and migration, as well as the formation of the extracellular matrix. Recent analyses revealed the overexpression of TGF-β1 in human gastric cancers and demonstrated increased cell proliferation in the stomach of patients with gastric cancer and their first-degree relatives. Using human gastric tissues obtained from patients with gastric cancer (*n* = 19), biopsies from healthy first-degree relatives of gastric cancer patients (*n* = 18) and healthy individuals (*n* = 19), we analysed the expression of TGF-β1 using the reverse transcriptase polymerase chain reaction (RT-PCR) and immunohistochemistry. Fifteen of 19 patients with gastric cancer expressed TGF-β1 in the tumour. In 11 of these 15 cases TGF-β1 mRNA was also detectable in the non-tumourous stomach. Interestingly, all but two individuals with a first-degree relative diagnosed with gastric cancer exhibited TGF-β1 expression in either the antrum or corpus biopsy or both. In contrast, only one of 19 individuals without a family history of gastric cancer expressed TGF-β1 in the stomach (*P*< 0.0001). TGF-β1 expression is detectable in a large proportion of gastric cancers and in the stomach of healthy first-degree relatives of gastric cancer patients. Since individuals without gastric cancers in their family express TGF-β1 only in one of 19 cases, the induction of TGF-β1 expression in first-degree relatives of patients with gastric cancer points to the presence of specific molecular alterations in a subgroup of individuals with an increased risk of developing gastric cancer that may precede the development of gastric cancers. © 2000 Cancer Research Campaign


*British Journal of Cancer* (2000) **82**(11), 1795–1800
© 2000 Cancer Research Campaign
DOI: 10.1054/ bjoc.1999.1107, available online at http://www.idealibrary.com on IDEAL®

### Expression of transforming growth factor beta-1 in gastric cancer and in the gastric mucosa of first-degree relatives of patients with gastric cancer

**MPA Ebert[1], J Yu[1], S Miehlke[4], G Fei[1], U Lendeckel[3], K Ridwelski[2], M Stolte[5], E Bayerdörffer[4] and P Malfertheiner[1]**

Departments of [1]Gastroenterology, Hepatology and Infectious Diseases, and [2]Surgery, [3]Institute of Experimental Internal Medicine, Otto-von-Guericke University Magdeburg, D-39120 Magdeburg, Germany; [4]Medical Department I, Technical University of Dresden, D-01307 Dresden, Germany; [5]Department of Pathology, Klinikum Bayreuth, D-95445 Bayreuth, Germany

**Summary** Transforming growth factors beta (TGF-β) constitute a family of polypeptide growth factors that control cell growth, cell differentiation and migration, as well as the formation of the extracellular matrix. Recent analyses revealed the overexpression of TGF-β1 in human gastric cancers and demonstrated increased cell proliferation in the stomach of patients with gastric cancer and their first-degree relatives. Using human gastric tissues obtained from patients with gastric cancer ($n$ = 19), biopsies from healthy first-degree relatives of gastric cancer patients ($n$ = 18) and healthy individuals ($n$ = 19), we analysed the expression of TGF-β1 using the reverse transcriptase polymerase chain reaction (RT-PCR) and immunohistochemistry. Fifteen of 19 patients with gastric cancer expressed TGF-β1 in the tumour. In 11 of these 15 cases TGF-β1 mRNA was also detectable in the non-tumourous stomach. Interestingly, all but two individuals with a first-degree relative diagnosed with gastric cancer exhibited TGF-β1 expression in either the antrum or corpus biopsy or both. In contrast, only one of 19 individuals without a family history of gastric cancer expressed TGF-β1 in the stomach ($P < 0.0001$). TGF-β1 expression is detectable in a large proportion of gastric cancers and in the stomach of healthy first-degree relatives of gastric cancer patients. Since individuals without gastric cancers in their family express TGF-β1 only in one of 19 cases, the induction of TGF-β1 expression in first-degree relatives of patients with gastric cancer points to the presence of specific molecular alterations in a subgroup of individuals with an increased risk of developing gastric cancer that may precede the development of gastric cancers. © 2000 Cancer Research Campaign

**Keywords:** risk; screening; carcinogenesis; stomach; family

Although the incidence of gastric cancer is declining, it remains the second most common cause of cancer related deaths in the world. The presence of advanced disease in the majority of patients with gastric cancer and the limited therapeutic options are responsible for the poor prognosis of patients suffering from gastric cancer (Fuchs et al, 1995; Delchier et al, 1998). Recently, molecular analysis has revealed the presence of various genetic and molecular alterations which contribute to the process of gastric carcinogenesis. Thus, activation of oncogenes, such as K-*sam* and c-*met*, as well as the inactivation of tumour suppressor genes, such as *p53*, have been reported to occur in gastric carcinoma (Wright et al, 1992; Tahara et al, 1996). Furthermore, alterations of adhesion molecules, such as mutation of the E-cadherin gene, have been reported in sporadic and familial gastric cancers (Tahara et al, 1996). Since histomorphological analysis has led to the hypothesis that specific histological changes of the gastric mucosa precede the development of gastric cancer, substantial efforts have been undertaken to reveal the molecular alterations underlying the histological changes, such as gastritis and intestinal metaplasia (Correa, 1992). However, the exact sequence of genetic and molecular alterations leading to the malignant transformation of the gastric mucosa remain largely unknown. Case–control studies have shown that family members of patients with gastric cancer have an increased risk of developing gastric cancer, and epidemiological analysis has revealed that the risk of developing gastric cancer is increased approximately threefold in these families (Macklin, 1960; Boeing et al, 1991; La Vecchia et al, 1992). Recently, Meining et al have demonstrated increased cell proliferation in the gastric mucosa of first-degree relatives of patients with gastric cancer irrespective of *Helicobacter pylori* infection (Meining et al, 1998).

Three mammalian isoforms of transforming growth factor beta (TGF-β) control the process of cell growth, cell differentiation, migration and the formation of the extracellular matrix (Massague et al, 1992). The biological activity of TGF-β1 is mediated through binding to a heteromeric complex of TGF-β receptors I and II (Massague et al, 1992; Kingsley, 1994). Recently, enhanced expression of TGF-β1 has been reported in human gastric cancers and TGF-β1 stimulates the fibrotic changes which occur in scirrhous gastric carcinoma (Mahara et al, 1994; Naef et al, 1997). Thus, TGF-β1 exerts its transforming potential mainly through the stimulation of stromal cells, angiogenesis and the suppression of immune surveillance. In an effort to further analyse the role of TGF-β1 in the process of gastric carcinogenesis, we assessed the expression of TGF-β1 in gastric cancers and the tumour-free gastric mucosa in patients with gastric cancer. Furthermore, in order to assess the role of TGF-β1 in the process of gastric carcinogenesis and its potential role in the progression of gastric precancerous lesions, we studied the expression of TGF-β1 in the gastric mucosa of first-degree relatives of patients with gastric cancer and healthy volunteers.

*Received 9 June 1999*
*Revised 20 December 1999*
*Accepted 20 December 1999*

*Correspondence to:* MPA Ebert

### MATERIALS AND METHODS

The following products were purchased: *Taq* polymerase from Gibco-BRL (Eggenstein, Germany). Oligonucleotides were purchased from Invitek (Berlin, Germany). All other chemicals and reagents were of molecular biology grade and were purchased from Sigma Chemical (Deisenhofen, Germany).

### Tissue samples

Normal and cancerous gastric tissue specimens were obtained from 19 patients undergoing upper intestinal endoscopy or gastric resection for gastric carcinoma. Ten males and nine females with a mean age of 62 years (range 35–73 years) were enrolled in this study. Biopsies were taken from the tumour and from the tumour-free corpus and antrum in nine patients undergoing upper gastro-intestinal endoscopy. In addition, a further group of patients ($n$ = 10) underwent gastric resection for gastric cancer. These tissues were obtained from the tumour and from the tumour-free gastric mucosa, which was located at least 8 cm from the margin of the tumours. Immediately following surgical or endoscopical removal all tissue samples were frozen in liquid nitrogen. Furthermore, 18 individuals with a first-degree relative who had undergone gastric resection for gastric cancer of the non-cardia type were consecutively recruited at three centres for upper gastrointestinal endoscopy. Four males and 14 females with a mean age of 47 years (range 27–65 years) were enrolled in this study. The patients' characteristics and histological diagnosis are given in Tables 1 and 4. Nineteen individuals with dyspeptic symptoms were recruited as controls. Patients with a malignancy, an active or a history of peptic ulcer, as well as a family history of malignant disease were excluded from the control group. All individuals gave informed consent to be included in the study. All studies were approved by the Human Subjects Committee of the Medical Faculty of the University of Magdeburg.

### Histological evaluation

Formalin-fixed biopsies were processed as previously described and sections were stained with haematoxylin and eosin, and with Warthin-Starry stain to detect *H. pylori* colonization. Grade and activity of the gastritis were determined in accordance with the Sydney system (Price, 1991). All histological assessments were performed by a trained pathologist who was blinded to the diagnosis and family history of the patient.

### RNA extraction

Total RNA was extracted from the human gastric tissue specimen by the acid guanidinium-thiocyanate method as previously described (Ebert et al, 1994).

### Polymerase chain reaction

Oligonucleotide primers were purchased from Invitek (Berlin, Germany). Primer sequences corresponding to human TGF-β1 mRNA were used for reverse transcriptase polymerase chain reaction (RT-PCR) analysis (Stratagene, Amsterdam, The Netherlands). β-Actin cDNA was amplified as previously described (Ebert et al, 1994) cDNAs were synthesized from total RNA (1 μg/sample) isolated from the gastric tissues, using oligodeoxythymidylate and reverse transcriptase (Ebert et al, 1994). Following inactivation, 1 μl of the reaction mixture was incubated in buffer containing 0.1 mM concentrations of dATP, dCTP, dGTP, dTTP, 0.2 μM concentrations each of oligonucleotide primers, 50 mM magnesium chloride ($MgCl_2$) and a 10× buffer consisting of 200 mM Tris–HCl (pH 8.0), 500 mM potassium chloride (KCl), and 1 U *Taq* polymerase. Reaction cycles consisting of 1.5 min at 95°C, 0.8 min at 60°C and 0.3 min at 72°C were repeated 35 times for amplification of human TGF-β1 (Massague et al, 1992; Kingsley, 1994). The PCR products were size fractionated on 1.25% agarose gels and visualized by ethidium bromide staining (Ebert et al, 1994). All PCR amplifications were performed at least twice. cDNAs reverse transcribed from mRNA extracted from mononucleate cells, were used as positive control (not shown). In order to confirm the specificity of the cDNA fragments generated by RT-PCR, we performed Southern blot analysis. The PCR fragments of five normal and five tumourous gastric tissues were size fractionated on 1% agarose gels, blotted on nylon membranes and hybridized by the method of Southern (Ebert et al, 1994).

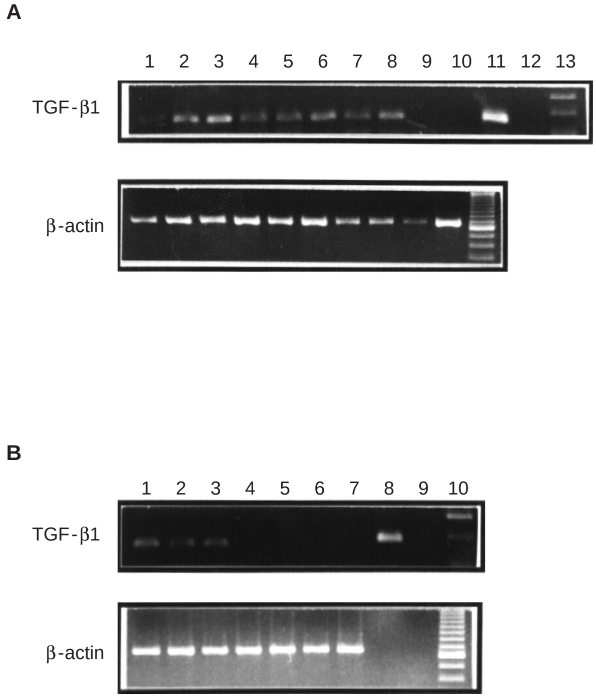

**Figure 1** RT-PCR analysis of TGF-β1 expression in gastric cancer and normal gastric mucosa. Using cDNAs obtained from total RNA which had been extracted from biopsies of gastric cancers and the tumour-free gastric mucosa, a band corresponding to TGF-β1 was present in 15 of 19 gastric cancers and in 16 of 18 gastric tissue specimens of first-degree relatives from patients with gastric cancer. In contrast, healthy volunteers with no family history of gastric cancer expressed TGF-β1 in only one case. (**A**) lanes 1–3: corpus, antrum, tumour biopsy of a patient with gastric cancer (Table 1, No. 8); lanes 4–6: corpus, antrum, tumour biopsy of a patient with gastric cancer (Table 1, No. 9); lanes 7–8: corpus and antrum biopsy of a first-degree relative (Table 4, No. 9); lanes 9–10: corpus and antrum biopsy of first-degree relative (Table 4, No. 10); lane 11: mononucleate cells serving as a positive control; lane 12: negative control; lane 13: DNA ladder. (**B**) Lanes 1–3: corpus, antrum, tumour biopsy of a patient with gastric cancer (Table 1, No. 6); lanes 4–7: normal control without a family history of gastric cancer; lane 8: mononucleate cells serving as a positive control; lane 9: negative control; lane 10: DNA ladder. Parallel PCR amplification of the cDNAs using two primers specific for β-actin confirmed the integrity of the cDNAs used

*© 2000 Cancer Research Campaign*

**Table 1** Patient characteristics and TGF-β1 expression in gastric cancer

| No. | Age | Sex | Hp | TGF-β1 expression | | Histological type | Lauren type |
|---|---|---|---|---|---|---|---|
| | | | | Tumour-free | Tumour | | |
| 1 | 65 | F | + | – | – | Adenocarcinoma | Diffuse |
| 2 | 69 | M | + | + | – | Adenocarcinoma | Diffuse |
| 3 | 71 | M | + | + | + | Adenocarcinoma | Intestinal |
| 4 | 55 | M | + | + | + | Signet ring cell | Diffuse |
| 5 | 60 | F | + | + | – | Signet ring cell | Diffuse |
| 6 | 62 | F | – | + | + | Adenocarcinoma | Intestinal |
| 7 | 73 | M | – | – | + | Small cell | |
| 8 | 69 | F | + | + | + | Adenocarcinoma | Diffuse |
| 9 | 37 | M | – | + | + | Adenocarcinoma | Intestinal |
| 10 | 63 | M | – | + | + | Adenocarcinoma | Intestinal |
| 11 | 69 | M | + | + | + | Adenocarcinoma | Diffuse |
| 12 | 63 | M | + | + | + | Adenocarcinoma | Intestinal |
| 13 | 69 | M | – | + | + | Adenocarcinoma | Intestinal |
| 14 | 66 | F | – | – | + | Signet ring cell | Diffuse |
| 15 | 35 | M | + | + | – | Signet ring cell | Diffuse |
| 16 | 57 | F | + | – | + | Signet ring cell | Diffuse |
| 17 | 64 | F | + | + | + | Adenocarcinoma | Intestinal |
| 18 | 54 | F | + | + | + | Adenocarcinoma | Intestinal |
| 19 | 63 | F | – | – | + | Signet ring cell | Diffuse |

F, female; M, male; –, negative; +, positive.

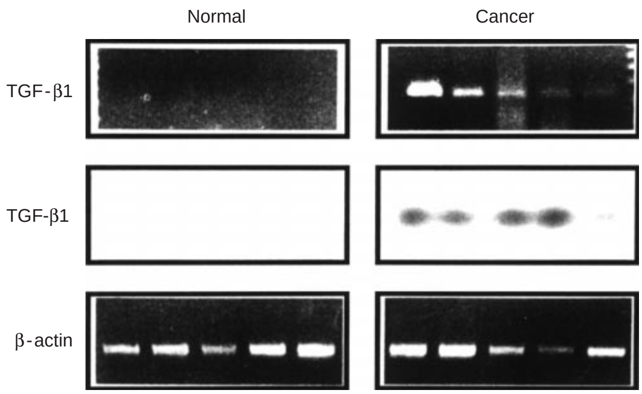

**Figure 2** Southern blot analysis. The specificity of the cDNA fragments corresponding to TGF-β1 was confirmed using Southern blot analysis as described in Methods. In the normal gastric mucosa TGF-β1 expression was not detected, however, in the gastric cancer tissues TGF-β1 was present as determined by Southern blot analysis of cDNA fragments generated by RT-PCR. All cases exhibited β-actin expression, confirming the integrity of the cDNAs used

### Immunohistochemistry

A highly specific polyclonal antibody against human TGF-β1 (Santa Cruz Biotechnology, Santa Cruz, CA, USA) was utilized for immunohistochemical analysis. The antibody was raised against an epitope corresponding to an amino acid sequence mapping at the carboxy terminus of the precursor form of human TGF-β1. This antibody is specific for TGF-β1, with no cross-reactivity with TGF-β2 or TGF-β3. Antibody specificity was confirmed by Western blotting and immunoprecipitation studies (Muso et al, 1996). Paraffin-embedded tissue sections from eight gastric cancers and six non-malignant stomachs were subjected to immunostaining using a streptavidin-peroxidase method. After blocking endogenous peroxidase activity with 0.3% hydrogen peroxide in methanol, the sections were incubated for 16 h with the anti-TGF-β1 antibody (1:100). Bound antibody was detected with a biotinylated IgG secondary antibody and streptavidin–peroxidase complex, using diaminobenzidine tetrahydrochloride as the substrate. Counterstaining was performed with Mayer's haematoxylin (Ebert et al, 1994).

### Statistical analysis

All data were analysed using the $\chi^2$ test, with $P < 0.05$ taken as the level of significance (Siegel, 1956).

### RESULTS

In the gastric mucosa of healthy controls TGF-β1 expression was present only in one of 19 cases (Figure 1). In these 19 cases biopsies were obtained from the corpus and antrum of patients with dyspeptic symptoms and histological analysis confirmed the diagnosis of gastritis in all cases. In contrast, 15 of 19 patients with gastric cancer exhibited TGF-β1 expression in the tumour as determined by RT-PCR analysis (Table 1). Southern blot analysis of the cDNA fragments encoding TGF-β1 confirmed the presence of TGF-β1 in the cancer samples (Figure 2). Using a highly specific anti-TGF-β1 antibody, TGF-β1 expression was observed in the gastric cancer cells by immunohistochemical analysis (Figure 3). The increased frequency of TGF-β1 in the gastric cancer tissues (15/19) differs significantly from the data obtained from the normal controls ($P < 0.0001$) (Table 2). In 14 of these 19 cancer patients, TGF-β1 expression was also observed in the tissue specimen obtained from the tumour-free stomach. Eleven cases co-expressed TGF-β1 in the gastric cancer and the tumour-free area (Table 1). However, in four cases TGF-β1 expression was only observed in the gastric tumour, whereas in three cases TGF-β1 was present in the tumour-free biopsy and was absent in the tumour. Interestingly, all gastric cancers which did not express TGF-β1 in the tumour were gastric cancers of the diffuse type

*© 2000 Cancer Research Campaign*

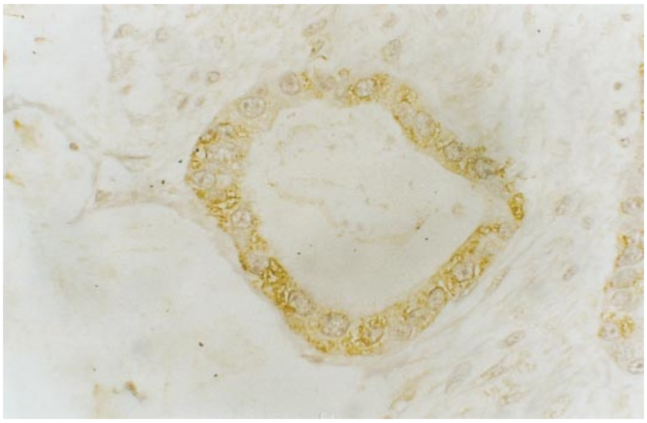

**Figure 3** Immunohistochemistry. Expression of TGF-β1 in gastric cancer cells was detected by immunohistochemical analysis. Gastric cancers of the intestinal type exhibited strong TGF-β1 immunoreactivity. Magnification: 400×

**Table 2** Statistical analysis of TGF-β1 expression in gastric biopsies of patients with gastric cancer and first-degree relatives versus normal controls

| | TGF-β1 expression | | *P*-value |
|---|---|---|---|
| | + | – | |
| Gastric cancer | 15 | 4 | 0.7085 |
| First-degree relative | 16 | 2 | |
| Gastric cancer | 15 | 4 | < 0.0001 |
| Healthy individual | 1 | 18 | |
| First-degree relative | 16 | 2 | < 0.0001 |
| Healthy individual | 1 | 18 | |

**Table 3** Statistical analysis of TGF-β1 expression in gastric biopsies of patients with gastric cancer and first-degree relatives

| | TGF-β1 expression | | *P*-value |
|---|---|---|---|
| | + | – | |
| Gastric cancer | | | |
| Tumour biopsy | 15 | 4 | 1.00 |
| Non-tumour biopsy | 14 | 5 | |
| Adenocarcinoma | 10 | 2 | 0.58 |
| Signet cell cancer | 4 | 2 | |
| Diffuse type | 6 | 4 | 0.09 |
| Intestinal type | 8 | 0 | |
| Hp positive | 8 | 4 | 0.24 |
| Hp negative | 7 | 0 | |
| First-degree relatives | | | |
| Corpus | 14 | 4 | 0.29 |
| Antrum | 10 | 8 | |
| No gastritis | 3 | 1 | 1.00 |
| Mild/moderate gastritis | 21 | 11 | |
| With intestinal metaplasia | 1 | 3 | 0.11 |
| Without intestinal metaplasia | 22 | 9 | |
| Hp positive | 10 | 1 | 1.00 |
| Hp negative | 6 | 1 | |

according to the Lauren classification, however, this finding did not reach statistical significance (Table 3). Furthermore, neither the histological type of gastric cancer, nor the status of *H. pylori* infection correlated with TGF-β1 expression in gastric cancer (Table 3).

In five patients with gastric cancer, a first-degree relative agreed to undergo upper gastrointestinal endoscopy in order to exclude gastric cancer and to determine the expression of TGF-β1 in biopsies obtained from the corpus and the antrum. Thirteen other individuals with a family history of gastric cancer were also studied.

**Table 4** Patients characteristics and TGF-β1 expression in gastric biopsies of first-degree relatives

| No. | Age | Sex | Hp | Corpus | | | Antrum | | | Intestinal metaplasia |
|---|---|---|---|---|---|---|---|---|---|---|
| | | | | Gastritis | | | Gastritis | | | |
| | | | | G | A | TGF-β1 | G | A | TGF-β1 | |
| 1 | 44 | F | + | 2 | 2 | + | 2 | 2 | + | – |
| 2 | 42 | F | – | 1 | 0 | + | 1 | 0 | + | – |
| 3 | 42 | F | – | 3 | 2 | + | 2 | 0 | – | – |
| 4 | 29 | M | + | 1 | 1 | + | 2 | 2 | + | – |
| 5 | 30 | F | – | 0 | 0 | + | 0 | 0 | – | – |
| 6 | 38 | M | + | 2 | 2 | + | 2 | 1 | – | – |
| 7 | 65 | M | + | 2 | 2 | + | 2 | 2 | + | – |
| 8 | 55 | F | + | 2 | 2 | + | 3 | 2 | – | + |
| 9 | 60 | F | + | 2 | 2 | + | 2 | 2 | + | – |
| 10 | 30 | F | – | 2 | 2 | – | 1 | 0 | – | + |
| 11 | 64 | F | + | 1 | 1 | – | 2 | 2 | + | – |
| 12 | 71 | F | + | 1 | 1 | + | 2 | 2 | + | – |
| 13 | 23 | F | + | 2 | 2 | + | 3 | 2 | – | + |
| 14 | 27 | F | – | 1 | 0 | + | 1 | 0 | + | – |
| 15 | 50 | F | + | 3 | 2 | – | 3 | 3 | + | + |
| 16 | 64 | F | – | 0 | 0 | + | 0 | 0 | + | – |
| 17 | 61 | F | + | 2 | 1 | – | 3 | 2 | – | – |
| 18 | 45 | M | – | 1 | 1 | + | ND | ND | – | – |

F, female; M, male; –, negative; +, positive; ND, not determined; G, grade of gastritis; A, activity of gastritis; level of gastritis: 0, no gastritis; 1, minimal; 2, moderate; 3, severe gastritis.

 *© 2000 Cancer Research Campaign*

We determined the expression of TGF-β1 in biopsies taken from the corpus and the antrum of these 18 individuals (Table 4). All but two of these 18 individuals exhibited TGF-β1 expression in either the biopsy taken from the corpus or the antrum or in both biopsies, a finding which contrasts with our results obtained from healthy individuals ($P < 0.0001$; Tables 2 and 4). Eight first-degree relatives co-expressed TGF-β1 in the corpus and antrum biopsy (Table 4). The presence of TGF-β1 in either the corpus or antrum biopsy did not correlate with the grade of the gastritis, *H. pylori* infection or the presence of intestinal metaplasia (Table 3).

### DISCUSSION

The pathogenesis of gastric cancer remains largely unknown. Epidemiological studies have revealed that *H. pylori* infection is associated with gastric cancer and, thus, *H. pylori* was classified as a group I carcinogen by the International Agency for Research on Cancer in 1994 (Fuchs et al, 1995). Further analysis revealed that *H. pylori* strains expressing the cagA gene may lead to more severe inflammation and may be more common in patients with gastric cancer. However, other studies could not find an association between cagA status and gastric cancer (Delchier et al, 1998). From histomorphological analysis a sequence of histological changes has been described which may precede the development of gastric cancer. Correa proposed that following atrophic gastritis, intestinal metaplasia, dysplasia and eventually gastric carcinoma may occur (Correa, 1992). However, the molecular alterations underlying these histological changes are largely unknown. The risk for the development of gastric cancer has been assessed using different histological criteria. Whereas the presence of intestinal metaplasia is still a matter of debate, other studies have shown that a corpus-dominant inflammation along with intestinal metaplasia in the antrum and corpus clearly defines a group of individuals with an increased risk of developing gastric cancer (Scott et al, 1990; Miehlke et al, 1997).

Molecular analysis has identified a number of genetic and molecular alterations which are present in gastric cancer. The activation of oncogenes such as the rearrangement of *TPR-MET*, leading to overexpression of the c-*met* protooncogene, as well as the amplification of K-*sam* have been reported in gastric cancers. In contrast, mutations of the K-*ras* oncogene are rare events in the process of gastric carcinogenesis (Soman et al, 1990; Tahara et al, 1996). Alteration of tumour suppressor genes such as *MCC* and *APC* occur in approximately one-third of gastric cancers, whereas *p53* gene mutations are present in the majority of gastric cancers (Nakatsuru et al, 1993). Recently, alterations of mismatch repair genes have been detected in gastric cancers. Ottini et al reported that the replication error positive phenotype is present in 30% of gastric cancers and that this phenotype is associated with an advanced tumour stage, intestinal type, and antral location (Ottini et al, 1997).

TGF-β1 binds to a heteromeric receptor complex of TGF-β receptors I and II (Massague et al, 1992) and activates signalling pathways which lead to the regulation of cell growth, cell differentiation and the production of the extracellular matrix (Kingsley, 1994). Various studies have shown that TGF-β1 is overexpressed in epithelial cancers and also in the stromal tissues surrounding the cancer cells. Among others, brain tumours, thyroid cancers and pancreatic cancers overexpress TGF-β1 in the tumour and the overexpression of this growth factor is associated with a poor survival in subgroups of these patients (Friess et al, 1993). Enhanced expression of TGF-β1 has also been reported in gastric cancers and in the fibrosis associated with scirrhous gastric carcinoma (Mahara et al, 1994; Naef et al, 1997). Furthermore, the expression of TGF-β1 in advanced gastric cancers is also associated with a shorter survival of these patients (Nakamura et al, 1998). The mechanism of TGF-β1 function in these cancers is believed to be mediated primarily by the stimulation of the extracellular matrix and suppression of an adequate immunological response. Recently, our group and others have demonstrated the induction of other growth factors in cancer cells by TGF-β1 which may be derived from the cancer cells or the surrounding stromal cells, pointing to autocrine and paracrine loops which give these cancer cells an additional growth advantage (Ebert et al, 1995). In our study, the expression of TGF-β1 in gastric cancers was assessed using both RT-PCR analysis and immunohistochemistry. For RT-PCR analysis, we used specific primers corresponding to TGF-β1 mRNA and performed RT-PCR analysis with tissue samples obtained during upper gastrointestinal endoscopy in patients with gastric cancer. We also used gastric tissues obtained from patients undergoing gastric surgery for gastric carcinoma. Tissues were obtained from the tumour itself and a tumour-free location. Our analysis demonstrates the expression of TGF-β1 in the vast majority of gastric cancers. The expression of TGF-β1 in the gastric cancer cells was confirmed by our immunohistochemical analysis. Only four tumours did not express TGF-β1 in the gastric tumour and all of these were of the diffuse type according to the Lauren classification. TGF-β1 was also detected in the tumour-free gastric mucosa in 14 of the 19 patients with gastric cancer. Since a few cancers did not exhibit TGF-β1 expression in the tumour, but expressed TGF-β1 in the tumour-free biopsy, we assume that the expression of TGF-β1 in these areas may result from the presence of inflammation in the tumour-free mucosa in these cases. The presence of TGF-β1 in either the tumour or the tumour-free tissue did not correlate with *H. pylori* infection, an observation which points to the presence of molecular mechanisms in gastric cancer which are independent of *H. pylori* and indicates that the role of *H. pylori* in gastric carcinogenesis may be rather confined to the induction of gastric inflammation.

Identifying individuals with an increased risk of developing cancers and the correct clinical management and follow-up of these individuals is a challenge for clinicians. Epidemiological studies have shown that first-degree relatives of patients with gastric cancer may have an increased risk of developing gastric cancer (Macklin, 1960; Boeing et al, 1991; La Vecchia et al, 1992). However, the genetic and molecular alterations underlying this increased risk remain largely unknown. Recently, increased cell proliferation in the gastric mucosa of individuals with a family history of gastric cancer has been demonstrated (Meining et al, 1998). This increase in cell proliferation was independent of *H. pylori* infection. The results of this study further support the hypothesis that not only environmental conditions, but most importantly, also genetic and molecular alterations contribute to the development of gastric cancer in first-degree relatives. We demonstrate a high frequency of TGF-β1 expression in gastric cancers and in the gastric mucosa of first-degree relatives, a finding which contrasts sharply with our data from healthy controls. Since TGF-β1 may mediate its transforming potential by indirect means, the presence of TGF-β1 in the gastric mucosa of first-degree relatives with an increased risk of developing gastric

*© 2000 Cancer Research Campaign*

cancer may indicate suppression of immune surveillance in these individuals which in turn may contribute to the progression of premalignant lesions in these individuals. In summary, our observations point to the presence of molecular alterations in the stomach of first-degree relatives which may precede the development of gastric cancers in this subgroup of individuals.

### ACKNOWLEDGEMENTS

This study was supported by grants from Land Sachsen-Anhalt (2775A/0087H) and the Deutsche Forschungsgemeinschaft (Eb 187/1–1, 1–2) awarded to MPA Ebert.

### REFERENCES

Boeing H, Frentzel-Beyme R, Berger M, Berndt V, Göres W, Körner M, Lohmeier R, Menarcher A, Männl HFK, Müller R, Ostermeier H, Paul F, Schwemmle K, Wagner KH and Wahrendorf J (1991) Case–control study on stomach cancer in Germany. *Int J Cancer* **47**: 858–864

Correa P (1992) Human gastric carcinogenesis: a multistep and multifactorial process – first American Cancer Society Award Lecture on cancer epidemiology and prevention. *Cancer Res* **52**: 6735–6740

Delchier JC, Ebert M and Malfertheiner P (1998) *Helicobacter pylori* in gastric lymphoma and carcinoma. *Curr Opin Gastroenterol* **14**: S41–S45

Ebert M, Yokoyama M, Kobrin MS, Friess H, Lopez ME, Büchler MW and Korc M (1994) Induction and expression of amphiregulin in human pancreatic cancer. *Cancer Res* **54**: 3959–3962

Ebert M, Yokoyama M, Friess H, Kobrin MS, Büchler M and Korc M (1995) Induction of platelet-derived growth factor A and B chains and overexpression of their receptors in human pancreatic cancer. *Int J Cancer* **62**: 529–535

Friess H, Yamanaka Y, Büchler MW, Ebert M, Beger HG, Gold LI and Korc M (1993) Enhanced expression of transforming growth factor β isoforms in pancreatic cancer correlates with decreased survival. *Gastroenterology* **105**: 1846–1856

Fuchs CS and Mayer RJ (1995) Gastric carcinoma. *N Engl J Med* **333**: 32–41

Kingsley D (1994) The TGF-β superfamily: new members, new receptors and new genetic tests of function in different organisms. *Genes Dev* **8**: 133–146

La Vecchia C, Negri E, Franceschi S and Gentile A (1992) Family history and the risk of stomach and colorectal cancer. *Cancer* **70**: 50–55

Macklin MT (1960) Inheritance of cancer of the stomach and large intestine in man. *J Natl Cancer Inst* **24**: 551–571

Mahara K, Kato J, Terui T, Takimoto R, Horimoto M, Murakami T, Mogi Y, Watanabe N, Kongo Y and Nutsu Y (1994) Transforming growth factor beta 1 secreted from scirrhous gastric cancer cells is associated with excess collagen deposition in the tissue. *Br J Cancer* **69**: 777–783

Massague J, Cheifetz S, Laiho M, Ralph DA, Weis FMB and Zentella A (1992) Transforming growth factor-β. *Cancer Surv* **12**: 81–103

Meining A, Hackelsberger A, Daenecke C, Stolte M, Bayerdörffer E and Ochsenkühn T (1998) Increased cell proliferation of the gastric mucosa in first degree relatives of gastric carcinoma patients. *Cancer* **83**: 876–881

Miehlke S, Hackelsberger A, Meining A, Hatz R, Lehn N, Stolte M and Bayerdörffer E (1997) Severe expression of corpus gastritis is characteristic in gastric cancer patients infected with *Helicobacter pylori*. *Int J Cancer* **73**: 837–839

Muso E, Yoshida H, Takeuchi E, Yashiro M, Matsushima H, Oyama A, Suyama K, Kawamura T, Kamata T, Miyawaki S, Izui S and Sasayama S (1996) Enhanced production of glomerular extracellular matrix in a new mouse strain of high serum IgA ddY mice. *Kidney Int* **50**: 1946–1957

Naef M, Ishiwata T, Friess H, Büchler MW, Gold LI and Korc M (1997) Differential localization of transforming growth factor-β isoforms in human gastric mucosa and overexpression in gastric carcinoma. *Int J Cancer* **71**: 131–137

Nakamura M, Katano M, Kuwahara A, Fujimoto K, Miyazaki K, Morisaki T and Mori M (1998) Transforming growth factor β1 (TGF-β1) is a preoperative prognostic indicator in advanced gastric carcinoma. *Br J Cancer* **78**: 1373–1378

Nakatsuru S, Yanagisawa A, Furukawa Y, Ichii S, Kato Y, Nakamura Y and Horii A (1993) Somatic mutations of the APC gene in precancerous lesions of the stomach. *Hum Mol Genet* **2**: 1463–1465

Ottini L, Palli D, Falchetti M, Dámico C, Amorosi A, Saieva C, Calzolari A, Cimoli F, Tatarelli C, De Marchis L, Masala G, Mariani-Costantini R and Cama A (1997) Microsatellite instability in gastric cancer is associated with tumor location and family history in a high-risk population from Tuscany. *Cancer Res* **57**: 4523–4529

Price AB (1991) The Sydney system: histological division. *J Gastroenterol Hepatol* **6**: 209–222

Siegel S (1956) *Non-parametric Statistics for Behavorial Sciences*. McGraw-Hill: New York

Scott N, Landsdown M, Diament R, Rathbone B, Murday V, Wyatt JI, McMahou M, Dixon M and Quirke P (1990) Helicobacter gastritis and intestinal metaplasia in a gastric cancer family. *Lancet* **335**: 728

Soman NR, Wogan GN and Rhim JS (1990) TPR-MET oncogenic rearrangement: detection by polymerase chain reaction amplification of the transcript and expression in human tumor cell lines. *Proc Natl Acad Sci USA* **87**: 738–742

Tahara E, Semba M and Tahara H (1996) Molecular biological observations in gastric cancer. *Semin Oncol* **23**: 307–315

Wright PA, Quirke P, Attanoos R and Williams GT (1992) Molecular pathology of gastric carcinoma: progress and prospects. *Hum Pathol* **23**: 848–859

*© 2000 Cancer Research Campaign*
